# Saphenous vein graft percutaneous coronary intervention: A different kind of animal

**DOI:** 10.1007/s12471-018-1074-8

**Published:** 2018-01-18

**Authors:** R. J. de Winter

**Affiliations:** Afdeling Cardiologie, B2-137, Academisch Medisch Centrum, Amsterdam, The Netherlands

In this issue of the Netherlands Heart Journal, IJsselmuiden et al. present a multicentre randomised control trial in which the STENTYS self-expanding bare metal stent (BMS) is compared with the STENTYS paclitaxel-eluting stent (PES) in saphenous vein grafts (SVGs). Primary endpoint of the trial was late lumen loss at six months follow-up, secondary endpoint was major adverse cardiac events (MACE) at 12 months. In a total of 57 patients (BMS 27 patients vs. PES 30 patients), the authors demonstrate that late lumen loss at 6 months was not significantly different (0.53 mm vs. 0.47 mm; *p* = 0.86) and MACE was comparable (BMS 22.2% vs. PES 26.7%).

The study is small and was hampered by slow enrolment, but with 57 patients and angiographic follow-up, the comparable late luminal loss is an acceptable and important result. The authors are to be commended for conducting and completing the study. The self-deploying STENTYS stent has several theoretical advantages for application in saphenous vein grafts, such as avoiding the risk of under- or oversizing because the self-deploying struts of the STENTYS stent will comply with the size of the graft. In addition, struts may be better apposed once the thrombus is dissolved and the risk of distal embolisation may be reduced because the stent deploys from distal to proximal. Distal embolisation was reported not to be present and the use of distal protection devices was at the discretion of the operators. It is unfortunate that the investigators were not able to perform intravascular imaging to confirm sizing and correct apposition of the stent struts after placement and at angiographic follow-up. Routine troponin measurements following the percutaneous coronary intervention (PCI) procedure were not reported. The main limitation of the study is its small sample size and the fact that only 70% of patients returned for angiographic follow-up despite all the efforts made by the investigators.

PCI of degenerated saphenous vein grafts has for a long time been a troublesome indication for PCI. Diffuse disease, long lesions, plaque shift during stent placement, distal embolisation of necrotic core and thrombus from the vessel wall, in-stent-restenosis and re-occlusion have been frustrating interventional operators for quite some time. The 2014 European guidelines for myocardial revascularisation recommend that

1. PCI should be considered as a first choice when technically feasible over re-do coronary artery bypass graft (class IIA LOE C);
2. PCI of the native bypassed artery is the preferred approach if technically feasible (class IIA, LOE C); and
3. that drug-eluting stents (DES) are recommended for PCI of saphenous vein grafts (class I LOE A) [1].

The guidelines state that ‘In the Swedish Coronary Angiography and Angioplasty Registry (SCAAR) of 3,063 procedures with 4,576 stents, including BMS and DES in saphenous vein graft lesions, the incidence of death was lower among patients who received DES’ [2]. However, no differences in terms of death, myocardial infarction or stent thrombosis were observed in the randomised ISAR-CABG (Is Drug-Eluting-Stenting Associated with Improved Results in Coronary Artery Bypass Grafts) trial [3].

In the study of IJsselmuiden et al. [4], the mean age of saphenous vein grafts was 16.2 years. We know that early graft occlusion after coronary artery bypass graft may be as high as 15% at one year. The best long-term revascularisation option for patients with multivessel disease may be avoiding the use of saphenous vein grafts altogether. Hybrid revascularisation may be the next best strategy [5]: fractional flow reserve/coronary flow reserve measurements of all coronary lesions, followed by arterial grafting using the left or right internal mammary artery and stenting of the lesions that cannot be reached with arterial grafts. There are some logistical issues, such as the question in what order to proceed: PCI first with CABG under dual antiplatelet or CABG first with PCI at a later point in time? Perhaps simultaneous surgical and interventional revascularisation in the hybrid operating room will solve this dilemma. Any strategy will likely trump the poor short-term and long-term fate of saphenous vein grafts, so this ‘different kind of animal’ will become extinct.
